# Radiculitis

**Published:** 1916-08

**Authors:** 


**Radiculitis.** In the Revue Ueurologique, Paris, March 1916, under the term radiculitis, Deyerine describes a syndrome consisting of sensory or sensori-motor symptoms in the distribution of the spinal nerves and which is produced by an inflammation of these roots within the meninges.

The most important clinical manifestation is pain of a neuralgic character, which is spontaneous and continuous with frequent exacerbations of violent character, and which are described by the patient as a tearing or breaking pain. The pains sometimes occur in the form of lancinating or so-called root pains.

These pains are not increased by movements nor by pressure on the muscles or nerve trunks as in nuritis, and, in the sciatic type of radiculitis the sign of Lasegue is also frequently absent.

Movements which exert traction on the roots themselves are very painful, such as inward rotation of the thigh when flexed on the abdomen. A characteristic sign is the exaggeration of the pain on coughing or sneezing, which is nearly always present during the early or inflammatory stage of the process.

Various paraethesias as tingling, numbness, heat and cold are frequently observed and these sensations have an accurate root distribution.

Objective disturbances of sensation as diminution or even complete loss may be found.

Motor disturbances are sometimes added to the sensory symptoms, constituting the sensori-motor type. These motor symptoms consist in weakness or even complete paralysis limited to the root areas involved. The paralysis is of the flaccid type and associated with muscular atrophy.

The disease may occur in various forms,—acute, sub-acute or chronic, depending on the rapidity and intensity of the inflammatory process.

The most frequent etiological factors of radiculitis are syphilis and tuberculosis. Cerebro-spinal meningitis and other infections involving the meninges, either directly or secondarily, may also produce it. According to Dejerine 80% of the cases are due to syphilis.

Pathologically the process is shown to be a localized meningitis surrounding the roots, or involving the meningeal sheath which accompanies the nerve roots. The meningeal sheath of the posterior root is always larger and deeper than that of the anterior root, and where it surrounds the root at the junction of the ganglion a point of least resistance is formed, and this is the reason for the predominance of the sensory symptoms over the motor disturbances. An important diagnostic point is obtained by lumbar puncture,—the spinal fluid showing a lymphocytosis, especially in recent cases.

Treatment. As most of the cases are of syphilitic origin the specific treatment is usually effective, especially the local treatment by intra-dural injections. In the non-syphilitic cases, Deperine recommends the local treatment with normal saline solution containing one-half percent of novocaine. In the severe cases which do not yield to any therapeutic measures, section of the posterior roots may be necessary to relieve the pain.—E. A. S.

